# Poisoning by Corrosive Sublimate

**Published:** 1844-11-30

**Authors:** 


POISONING BY CORROSIVE SUBLIMATE.

Dr. Watson, of the Royal Infirmary, at Glasgow, records in the London and Edinburgh Medical Journal, a case of poisoning by corrosive sublimate, which occurred in his practice, the principal features of which were that, although the patient lived seven days after having swallowed the poison, no real salivation took place. The gums were only slightly swollen; there was no mercurial fœtor; the teeth were firm; and the flow of saliva was confined to the first day after admission into the hospital, when it might be partly occasioned by nausea, but especially by the irritation of the fauces, and difficulty of swallowing. The effects of the poison were confined to the alimentary canal, or nearly so, but the whole track of the canal was not equally acted on: the œsophagus, the left half of the stomach, the lower part of the ileum, the colon, and especially the rectum, were chiefly affected; and those parts, although the seat of violent action, were free from abrasion. The urinary bladder, and the lining membrane of the larger bronchi, had also participated, although in a minor degree, in the general irritation of the mucous membrane. Chemical analysis did not detect any traces of the poison in the alimentary canal. The liver, in which Orfila has recently discovered indications of corrosive sublimate in cases where it has been administered internally, does not appear to have been examined chemically.—*Lond. Med. Times.*

